# Clinical Manifestations in Vacuoles, E1 Enzyme, X-Linked, Autoinflammatory, Somatic (VEXAS) Syndrome: A Narrative Review

**DOI:** 10.7759/cureus.53041

**Published:** 2024-01-27

**Authors:** Vlad Padureanu, Cristian Marius Marinaș, Anca Bobirca, Rodica Padureanu, Stefan Patrascu, Ana Maria Dascalu, Florin Bobirca, Laura Tribus, Cristina Alexandru, Crenguta Serboiu, Catalin Dumitrascu, Anca Musetescu

**Affiliations:** 1 Department of Internal Medicine, Emergency County Hospital of Craiova, University of Medicine and Pharmacy of Craiova, Craiova, ROU; 2 Department of Anatomy, University of Medicine and Pharmacy of Craiova, Craiova, ROU; 3 Department of Internal Medicine and Rheumatology, Carol Davila University of Medicine and Pharmacy, Bucharest, ROU; 4 Department of Internal Medicine, University of Medicine and Pharmacy of Craiova, Craiova, ROU; 5 Department of Surgery, University of Medicine and Pharmacy of Craiova, Craiova, ROU; 6 Department of Ophthalmology, Carol Davila University of Medicine and Pharmacy, Bucharest, ROU; 7 Department of General Surgery, Carol Davila University of Medicine and Pharmacy, Bucharest, ROU; 8 Department of Gastroenterology, Faculty of Oral Medicine, Carol Davila University of Medicine and Pharmacy, Bucharest, ROU; 9 Department of Histology, Cellular and Molecular Biology, Carol Davila University of Medicine and Pharmacy, Bucharest, ROU; 10 Department of Rheumatology, University of Medicine and Pharmacy of Craiova, Craiova, ROU

**Keywords:** recognize, diagnose, clinical manifestations, syndrome, vexas

## Abstract

The newly identified refractory adult-onset autoinflammatory syndrome known as VEXAS (vacuoles, E1 enzyme, X-linked, autoinflammatory, somatic) syndrome is brought on by somatic mutations in the ubiquitin-like modifier-activating enzyme 1 (UBA1) gene in hematopoietic stem and progenitor cells that change the expression of the UBA1 isoform. As a result, patients have a variety of hematologic and systemic inflammatory symptoms. All types of medical professionals should treat VEXAS syndrome seriously due to the high fatality rate. To better comprehend the condition and enhance the prognosis for VEXAS syndrome, this review article describes the essential traits and clinical signs of the condition. The discussion of future directions in the study of systemic inflammatory disorders brought on by somatic mutations concludes.

## Introduction and background

VEXAS (vacuoles, E1 enzyme, X-linked, autoinflammatory, somatic) syndrome is a recently discovered category of autoinflammatory diseases, first described in 2020 by Beck et al. [1]. The authors found a cohort of 25 male patients with systemic inflammatory symptoms in combination with hematological ones, having a somatic mutation in ubiquitin-activating enzyme 1 (UBA1) gene. The peculiar aspect of this new autoinflammatory syndrome is the fact that the mutation is acquired later in life, with the target population being males.

Ubiquitin-activating enzyme, E1 enzyme, encoded by UBA1 gene is responsible for the initiation of the ubiquitylation process leading to intracellular degranulation. The underlying pathogenesis of VEXAS syndrome is UBA1 mutations in hematopoietic stem cells and myeloid-lineage cells, by substitution of methionine-41 (pMEt41), consequently decreasing the breakdown of proteins, disruption of cell homeostasis, and increased endoplasmic reticulum stress [2]. The direct consequence of this process is the activation of autoimmune pathways, which in turn causes an uncontrolled inflammatory reaction [1,3,4].

A retrospective observational study by Beck et al. found that out of the 163 096 participants (mean age, 52.8 years; 94% White; 61% women), 11 individuals had probable somatic mutations with known pathogenic UBA1 variants, all of which had clinical manifestations typical of VEXAS syndrome (nine males and two females) [5].

VEXAS patients have a vast multiorgan clinical spectrum, and the diagnosis is more frequent after the age of 40 years old, which suggests a late disease onset or inadequate diagnosis in the early stages being sometimes difficult to exclude other rheumatological systemic diseases or hematological disorders [6]. The VEXAS condition also imparts an important lesson to clinicians who regularly deal with "outliers," or individuals who contradict our present knowledge of physiology.

Vacuoles

The presence of cytoplasmic vacuoles in myeloid precursor cells is one of the VEXAS characteristics. Eosinophils, monocytes, megakaryocytes, and plasma cells can also occasionally have vacuoles, although mature lymphocytes and fibroblasts typically don't, which may be connected to the location of the UBA1 somatic mutation [1,7,8]. Cytoplasmic vacuoles are not a particular diagnostic requirement in order to identify VEXAS syndrome, as they can also be present in other diseases including acute alcoholism, zinc toxicity, copper insufficiency, and myelodysplastic syndrome (MDS) [9-11]. With great sensitivity and specificity, Lacombe et al. determined that VEXAS syndrome was associated with the threshold of 10% neutrophil precursors with more than one vacuole [3].

E1 enzyme

The ubiquitin-activating enzyme (E1 enzyme), which is essential for cellular ubiquitylation, is encoded by the UBA1 gene [12]. The process of ubiquitination, which uses the enzymes ubiquitin-activating (E1), conjugating (E2), and ligating (E3) in succession, is used to degrade misfolded proteins. Numerous biological functions, including the response to DNA damage, cell cycle progression, and immunological signaling pathways, depend on ubiquitylation [10]. Reduced ubiquitination and the activation of autoimmune pathways brought on by UBA1 mutations result in inflammatory symptoms [1,3,12,13].

X-Linked

UBA1 mutations can be avoided through X chromosome inactivation because the gene is situated on the X chromosome (Xp11.3). The fact that elderly men are more likely to develop VEXAS syndrome suggests that women may be shielded through the unaltered allele [1,14-16]. However, a small number of female individuals with acquired X chromosomal monosomy or X chromosome structural deletion have been documented [17,18].

Autoinflammatory

Ubiquitylation defects play a definite role in diseases with enhanced systemic inflammation, the compromised step of the ubiquitylation machinery dictating the effect on cell stress and activation of autoimmunological pathways [2]. Uncertainty exists regarding the mechanism by which UBA1 abnormalities cause a variety of autoinflammatory symptoms. Fever, nose and ear chondritis, dermatological symptoms, vasculitis, and pulmonary infiltrates, are examples of multiorgan autoinflammatory signs. The patient's peripheral blood transcriptomic study further demonstrated the activation of numerous innate immune pathways [1,19]. Beck et al. discovered elevated gene expression of proinflammatory cytokines, indicating that systemic inflammatory symptoms in individuals with VEXAS syndrome may be related to UBA1 mutations [1].

Somatic

Since the UBA1 mutation in VEXAS syndrome is a somatic mutation, not all myeloid cells have it. By Sanger sequencing, performed on either blood marrow or blood samples, somatic mutations can be detected down to a minimum of 15% to 20% [20]. High variant allele frequencies (VAF) of UBA1 discovered by exon sequencing, however, have been reported to be mistaken for germline mutations connected to X-linked spinal muscular atrophy 2 (SMAX2), which may prejudice the diagnosis or potentially have an impact on future treatment [21]. When the patient's clinical appearance is inconsistent with SMAX2, sequencing of other tissues may be taken into account.

## Review

Material and methods

Applying the terms "clinical manifestations" and "VEXAS syndrome," 88 articles were found in the PubMed databases. During the research, letters, remarks, and opinions were not taken into consideration, only the most relevant articles were included in order to realize a comprehensive narrative review of VEXAS clinical manifestations.

Clinical manifestations

**Figure 1:**
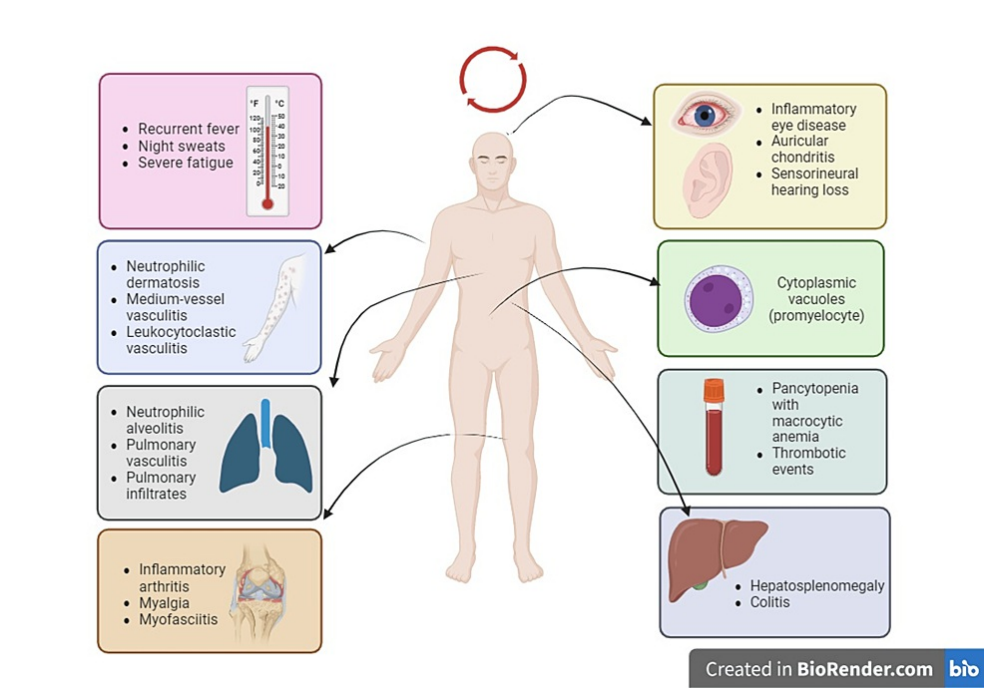
Clinical manifestations in VEXAS Figure created by the authors in BioRender.com VEXAS: Vacuoles, E1 enzyme, X-linked, autoinflammatory, somatic

VEXAS syndrome is a debilitating and progressive disorder characterized by the simultaneous involvement of many organ systems, as can be observed in Figure 1. The primary clinical manifestations of this syndrome predominantly consist of systemic inflammatory symptoms and hematological abnormalities. Since its initial documentation in 2020, the range of clinical presentations has progressively broadened due to the exponential rise in reported instances of VEXAS.

Constitutional symptoms

In several large cohorts that have been published, 74% of patients had recurrent fever, which is the most prevalent presentation. Additional discomforts including weariness, poor appetite, and weight loss may accompany it, and increased acute-phase reactants may be detected [21]. It could be the first sign of VEXAS or a side effect of receiving repeated immunosuppressive treatments that results in an acquired immunodeficiency [22].

Dermatological symptoms

Dermatologists ought to become comfortable with the clinical presentation because cutaneous involvement is frequently the first indicator of disease [23]. Skin symptoms were also among the most frequent presentations, reported around 83-88% [1,24]. The skin lesions occur in cycles and generally disappear after 7-10 days, reoccurring at random over time, from 2 up to 10 times annually [25]. Non-vasculitic lesions (such as Sweet’s syndrome, neutrophilic dermatosis, pressure plaques, erythema nodosum, urticaria, injection site reactions, insect bites reactions, subcutaneous nodules, lupus tumidus, panniculitis, livedo reticularis, periorbital edema, and others) and vasculitic lesions (such as leukocytoclastic vasculitis) cutaneous manifestations are the most frequently encountered [23,26]. Mixed cryoglobulinemia has been reported to cause secondary skin sequelae as a result of necrosis [27].

The cutaneous lesion presentation of VEXAS syndrome has broadened to encompass a greater range of morphologies, including urticarial, papular, nodular, eczematous, targetoid, and polycyclic, as additional data has been gathered since the condition was first described in 2020 [21,24,28-35]. There have also been reports of uncommon morphologies, including joint contractures and extensive fibrosis after an episode of significant peripheral edema [36], desquamative morbilliform eruptions [37], urticarial lesions associated with lymphadenitis, and cutaneous papules that mimic Kikuchi-Fujimoto disease both histologically and clinically [29,38-40]. The skin biopsy could represent an important tool in order to establish the diagnosis. Usually, the lesions are superficial, being described as infiltrates of immature myeloid cells, neutrophils, lymphocytes, histiocytes, and interstitial edema, commonly leukocytoclastic vasculitis and rarely neutrophil urticarial dermatosis [41].

Using sequencing and analysis, Zakine et al. found the UBA1 mutation in skin samples from VEXAS patients. Consequently, rather than an inflammatory backdrop, the scientists determined that the skin penetration appeared to be dependent on the clonal growth of UBA1 mutant cells [25,42]. It is possible that UBA1 mutant clones are absent from non-neutrophilic dermatitis or that there is not enough of their clone to be noticed, a theory supported by Lacombe et al. who discovered that neutrophilic dermatosis patients' skin tissues contain UBA1 mutations, but not those from other dermatoses (cutaneous leukocyte fragmentation vasculitis and spacer lipofuscinosis) [43]. VEXAS must be included in the dermatologist's list of differential diagnoses when verifying skin condition, since patients may initially be seen for skin lesions. In male patients over 50 years old presenting with common VEXAS dermatological manifestations such as leukocytic vasculitis or neutrophilic dermatosis and macrocytic anemia, genetic testing should be considered.

Relapsing polychondritis (RP)

More than half of the patients met the diagnostic criteria for RP prior to establishing the diagnosis of VEXAS, usually they have ear and nasal cartilage involvement [26]. According to Khitri et al., the VEXAS-RP group had a worse prognosis, greater death rate, older patients, a lower rate response to medication, and a higher frequency of skin lesions (82%), fever (58%), lung infiltrates (29%), and myelodysplastic syndrome (75%) [44]. The accuracy of VEXAS diagnosis needs genetic testing for UBA1 mutation, the same authors excluded from the genetic evaluation the patients older than 65 years old, with platelets count <200 k/μL and mean corpuscular volume >100 fL, which are more suitable for the primary RP. Georgin-Lavialle et al. in their multicentric study discovered a frequency of 36% of relapsing chondritis among 166 VEXAS patients [24].

The mouth and genital ulcers with inflamed cartilage (MAGIC) condition may be initially diagnosed as a result of arthritis linked with genital and oral ulcers [45]. Tracheobronchial cartilage involvement has also been reported [46]. A crucial clue for diagnosing VEXAS syndrome in patients presenting with chondritis is the presence of macrocytic anemia [29].

Systemic vasculitis

Vasculitis affecting small, medium, and large blood vessels can all be brought on by VEXAS syndrome. Giant cell arteritis, a large vessel vasculitis, was identified in 1 out of 25 patients (4%), according to Beck [1]. Immunoglobulin A vasculitis with small vessel vasculitis and antineutrophil cytoplasmic antibody-associated vasculitis have been documented with rare incidences like medium vessel vasculitis with polyarteritis nodosa [10]. It is worth mentioning that even though cutaneous morphology can present as polyarteritis nodosa, VEXAS patients typically lack mesenteric vasculitis [1,30]. Regarding small vessel vasculitis and VEXAS-related vasculitis, both are characterized by abnormal neutrophil activation with increased NET formation [47].

Even though the involvement of all vessel sizes has been described, cutaneous small vessel vasculitis seems to be the most common form [1,18,24,27,48]. The increasing number of cases that are being reported indicates that VEXAS should be taken into consideration as a differential diagnosis for refractory vasculitis that has aberrant hematological symptoms [49].

Lung involvement

Respiratory manifestations are habitually described in VEXAS syndrome, usually with good outcomes at corticotherapy administration, seen in some studies in 49% of cases [24,50]. Up to 91% of patients with VEXAS syndrome have lung involvement, including pleural inflammation, neutrophilic alveolitis, cryptogenic organizing pneumonia, nonspecific interstitial pneumonia, bronchiolitis obliterans, and mediastinal lymphadenopathy. On chest computed tomography pleural effusions and widespread bilateral ground-glass opacities were seen [1,10,22]. The pulmonary involvement could be considered an advanced stage of disease, a systemic one, with extensive organ involvement, that requires immunosuppressive drugs and corticotherapy in variable dosages [50].

Myelodysplastic syndromes

In VEXAS patients, bone marrow is regularly affected with an increased association of a hematological disorder such as myelodysplastic syndrome (MDS) or even neoplasms. Cytopenia is frequently diagnosed in VEXAS patients, but the exact pathological mechanism is yet to be established, certainly, the increased levels of inflammatory cytokines (like interferon gamma and interleukin 8) due to the disruption of normal UBA1 activity is playing a central role [4]. The other most common hematological abnormalities are macrocytic anemia, bone marrow hypercellularity, and dysplasia [8].

Several research cohorts are describing patients with VEXAS syndrome and concomitant MDS. In a multicentric French study, including 116 VEXAS patients, MDS was diagnosed in 49% [24]. Moreover, in this study there was reported a mortality rate of 15.5% at a median follow-up of 3 years, 3/18 being due to MDS progression. There is no clear evidence on the type of relationship between VEXAS syndrome and progressive MDS, but the current theories indicate that changes in the hematological microenvironment could play a central role [51]. According to one study, myelodysplasia, macrocytic anemia, and pancytopenia were seen in patients whose severe systemic inflammation increased, but not when their symptoms subsided. This suggests that VEXAS syndrome-related cytopenia and bone marrow dysplasia are linked to systemic inflammation [52]. Some VEXAS patients have been shown to have DNMT3A gene mutations, which could be the cause of the coexistence of hematologic malignancies like MDS in VEXAS syndrome patients [13,52,53].

Lymphopenia can appear in the course of the disease, either due to incompatibility with survival in mature cells with UBA1 mutations or as a result of corticoid treatment [54].

Additionally, it has been proposed that the closely linked DNMT3A mutation to MDS may aid in the amplification of VEXAS clones and possibly even cause more severe inflammatory symptoms, as previously indicated. It is still not possible to determine the cause and effect [15,28]. Due to their increased susceptibility to MDS and other related hematologic malignancies due to the UBA1 mutation, these patients are more likely than others to need close clinical follow-up [54].

Thrombotic events

The French multicentric study reported 35% of patients with venous thrombosis as a VEXAS manifestation [24]. Early on in VEXAS, thrombotic events could happen. In the initial report of VEXAS syndrome, 11 out of 25 patients (44%), had venous thromboembolism [1]. According to Obiorah et al., 10 out of 16 patients (63%) had a greater incidence of thrombotic problems, and a tiny number of patients experienced a relapse while receiving anticoagulant medication [7]. Venous thrombosis was far more common than arterial thrombosis, with an incidence of 34.4 and 1.6%, respectively [55]. The presence of pathological antiphospholipid antibodies, prolonged inflammation, abnormal cytokine release, increased spontaneous NET formation, and abnormal hemostasis are some of the factors that can lead to abnormal hemostasis, platelet activation, endothelial cell dysfunction, and even possibly the coagulation cascade [56]. Dysregulation of ubiquitination caused by UBA1 somatic mutations has been proposed as a major driver of VEXAS thrombosis [8,55]. This implies that immunosuppression and immunomodulation could be used as a tactic to stop thrombotic episodes. Thrombocytopenia, hemophagocytic lymphohistiocytosis, plasma cell disease, macrophage activation syndrome, systemic AA amyloidosis, progressive bone marrow failure, and chronic lymphocytic leukemia are additional hematologic illnesses that can affect people with VEXAS syndrome. However, there is no established causal connection between VEXAS and these illnesses [37,57].

Other clinical manifestations

Additional infrequent clinical presentations have been gradually documented. These comprise ocular inflammation (uveitis, periorbital edema, scleritis), gastrointestinal infiltration (persistent diarrhea, abdominal pain, gastrointestinal bleeding), arthritis (primarily affecting small joints of the hand, ankle, and knee) and other musculoskeletal afflictions such as rheumatoid arthritis-like severe erosive disease, myalgia and myofasciitis, enlargement of lymph nodes, cardiac involvement (pericarditis, myocarditis), and neurological involvement (sensory neuropathy, hearing loss, acute attacks of chronic inflammatory demyelinating polyneuropathy, and even involvement of the central nervous system) [22,28,58,59]. Even though the nature of the relation is uncertain, a case of endocarditis possibly associated with VEXAS has been reported among patients with chondritis in a Japanese cohort [21].

A VEXAS patient with concomitant ANCA-vasculitis was described in a single-center Italian cohort research. Upon renal biopsy, the patient had a distinctive presentation of necrotizing and crescentic glomerulonephritis [48]. The clinical manifestations of VEXAS syndrome have been linked to specific mutant genotypes, as more and more cases are documented. These genotypes are P. Met41Val for an unexplained inflammatory condition, P. Met41Thr for inflammatory ocular disease, and P. Met41Leu for Sweet syndrome. P.Met41Val was shown to have a greater death rate and a much shorter median survival time in a cohort study compared to other mutations [28,52]. The diagnosis of VEXAS should be taken into consideration in all patients with increasing hematologic abnormalities and refractory inflammatory illness, as early recognition and diagnosis of the condition might be difficult.

Prognosis

According to the clinical data that is currently available, patients with VEXAS have high rates of clinical heterogeneity and mortality, ranging from 20 to 49% in different cohorts. These patients may also have a poor prognosis because of delayed diagnosis and recognition, hematologic disease progression, and a lack of effective and efficient treatment options. Patients may pass away from illnesses or consequences of their treatments. Risk factors for increased mortality include lung infiltration, expansion of the mediastinal lymph nodes, transfusion dependency, and gastrointestinal involvement [28]. The French research cohort's results, which suggested that patients with P. Met41Leu may have a better prognosis, may not be consistent with the P. Met41Val mutant genotype, which was also found to be a poor prognostic factor [24]. This discrepancy could be attributed to the latter study cohort's shorter follow-up period. It's possible that the variations in residual UBA1b translation amongst the mutants account for the differences in survival rates [26]. In this study group, there is no correlation seen between the UBA1 VAF level and illness severity or mortality. Therapeutic effectiveness is still poor for VEXAS patients, corticosteroids being the first line of treatment. However, new accruing data has shown that JAK-inhibitor treatment and anti-IL6 therapy are an efficient therapeutic option and should be taken into consideration [60-62].

## Conclusions

It has been recently discovered that VEXAS syndrome is a refractory adult-onset inflammatory illness that primarily affects older men. Systemic inflammation and hematologic abnormalities are the most common symptoms, and while high-dose glucocorticoids are one of the few known therapies, new therapies such as biological therapy and JAK inhibitors are showing promise.

It may be useful to target and eliminate UBA1 mutations (using UBA1 gene editing to change mutant clones or HSCT to replace mutated progenitor cells), to stop the inflammatory cascade response (focusing on the ubiquitination pathway), or even to think about restoring the function of UBA1b, depending on the understanding of the molecular basis of the disease. Gene editing treatments and bone marrow transplants could be treatment options. Reducing the amount of time between the development of VEXAS syndrome symptoms and genetic diagnosis permits the early evaluation of allogeneic bone marrow transplantation. In order to help with the early diagnosis of the syndrome and to enhance prognosis, testing for UBA1 mutations is therefore advised for adult patients with hematologic abnormalities, systemic inflammation, and vacuolation of erythroid and myeloid precursor cells.
